# Association between the CALLY index, vitamin D, and asthma: insights from NHANES

**DOI:** 10.3389/falgy.2025.1557677

**Published:** 2025-04-07

**Authors:** Yachun Liu, Yufeng Wei

**Affiliations:** Department of Pharmacy, Ganzhou People's Hospital, Ganzhou, Jiangxi, China

**Keywords:** vitamin D, CALLY, asthma, National Health and Nutrition Examination Survey (NHANES), mediated effects

## Abstract

**Purpose:**

The CALLY index integrates C-reactive protein (CRP), albumin and lymphocyte counts to accurately reflect the inflammatory, nutritional and immune status of the body. Multiple studies have indicated that the CALLY index plays a key role in a variety of diseases, especially asthma, and is closely associated with inflammatory response, airway remodeling and immune imbalance in asthma. Research has shown that vitamin D is associated with asthma susceptibility, severity, and control, and its levels may influence inflammatory and immune markers associated with the CALLY index, which may play a role in the association between the CALLY index and asthma.

**Patients:**

Using data from the National Health and Nutrition Examination Survey (NHANES) from 2001 to 2010, the association between CALLY index and asthma and the role of vitamin D in American adults were analyzed in depth. Through multiple logistic regression, subgroup analysis, and other statistical means, the potential pathophysiological links between the three are revealed, providing theoretical support for the prevention and treatment of related diseases.

**Results:**

A total of 17,946 individuals were included in this study, of which 2,317 were diagnosed with asthma. Fully adjusted multivariate logistic regression analysis revealed that the CALLY index was significantly negatively associated with asthma, with an odds ratio (OR) of 0.996 [95% confidence interval (CI) 0.993–0.999]. Specifically, each unit increase in the CALLY index was associated with a 0.996-fold reduction in asthma risk. In addition, mediation effect analysis showed that vitamin D partially mediated the association between the CALLY index and asthma, with a mediation ratio of 3.36%.

**Conclusion:**

This study reveals an association between the CALLY index and reduced risk of asthma in the US population and suggests that vitamin D plays an incomplete mediating role. This finding provides a new theoretical basis for the diagnosis, treatment, and prevention of asthma and is expected to be a potential biomarker.

## Introduction

In recent years, many studies have focused on the role of systemic inflammatory markers and immune status in asthma, among which the C-reactive protein-albumin-lymphocyte (CALLY) index, as a comprehensive indicator reflecting the body's inflammation and immune status, has received increasing attention for its association with asthma. The CALLY index integrates C-reactive protein (CRP), albumin and lymphocyte counts to accurately reflect the inflammatory, nutritional and immune status of the body. Multiple studies have indicated that the CALLY index plays a key role in a variety of diseases, especially Asthma, and is closely associated with inflammatory response, airway remodeling and immune imbalance in asthma (1). Meanwhile, vitamin D not only plays a key role in calcium and phosphorus metabolism but also participates in the regulation of the immune system. In addition, its association with asthma has also become a hot research topic.

Research has shown that Vitamin D is associated with Asthma susceptibility, severity, and control (2), and its levels may influence inflammatory and immune markers associated with the CALLY index, which may play a role in the association between the CALLY index and asthma (3). As a common chronic respiratory disease, the global prevalence of asthma is rising, bringing a heavy burden to patients and society (4). According to relevant statistics, the number of asthma patients worldwide is approximately 300 million, and the number of new cases is increasing every year, especially in children and adolescents. Asthma is essentially a chronic disease of the airways and has long been viewed primarily as an inflammatory disease; therefore, current treatments focus on inflammation management (5).

The CALLY index is a composite index that incorporates C-reactive protein (CRP), albumin, and lymphocyte count. CRP is an acute phase response protein that is synthesized primarily by the liver. However, in asthmatic airways, local production of CRP also occurs. Studies have shown that airway epithelial cells and inflammatory cells in asthma patients can produce CRP locally (6, 7). Local inflammation in the airways can activate signaling pathways in these cells, leading to increased CRP synthesis. This local CRP can directly participate in the inflammatory response in asthmatic airways by activating the complement system, promoting the adhesion and phagocytosis of inflammatory cells, and aggravating airway inflammation and damage. Therefore, CRP in asthmatic airways has both systemic and local effects. CRP rises rapidly in response to inflammatory stimuli. Studies have shown that CRP is able to exacerbate the inflammatory response in asthmatic airways by activating the complement system and promoting adhesion and phagocytosis of inflammatory cells. In addition, CRP is strongly associated with the frequency and severity of asthma attacks, and serum CRP levels are negatively correlated with the severity of acute asthma attacks (8). Albumin, a protein abundant in plasma, is crucial for physiological functions like maintaining colloid osmotic pressure and transporting substances. Its levels can mirror an organism's nutritional state. T lymphocytes are accountable for airway inflammation and hyperreactivity in asthma patients, whereas B lymphocytes participate in the allergic reaction process in asthma. The CALLY index has demonstrated significant value in various diseases, and its influence on respiratory diseases might be associated with the inflammatory responses in asthma. Specifically, the CALLY index is of importance in multiple diseases, and its impact on respiratory diseases may be closely linked to the inflammatory response, airway remodeling, and immune imbalance in asthma.

Vitamin D has been well-documented to assume a crucial position in calcium and phosphorus metabolism. Beyond that, it is implicated in diverse physiological processes, among which immunomodulation is included. An increasing quantity of research has revealed a connection between vitamin D levels and the susceptibility to, severity of, and control over asthma. Furthermore, considering its association with inflammatory and immune markers, these studies imply that vitamin D might play a potential mediating role in the relationship between the CALLY index and asthma. To date, no research has been reported elucidating the association between the CALLY index and asthma, nor has there been any exploration into whether the influence of the CALLY index on asthma is mediated by vitamin D levels. An in-depth investigation of this relationship holds significant clinical importance. Aiming to fill this void in knowledge, the present study endeavors to conduct a large-scale cross-sectional survey by leveraging data from the National Health and Nutrition Examination Survey (NHANES) carried out between 2001 and 2010. The objective of the present study was to clarify the association between the CALLY index and asthma, as well as the mediating role played by vitamin D levels within this association.

## Materials and methods

### The design of the study and the individuals involved

The NHANES is a research project that utilizes a complex sampling design to evaluate the health and nutritional conditions of the US population. It gathers basic information, questionnaire responses, physical examination outcomes, and various laboratory test data from the participants. The survival status of NHANES participants as of 31 December 2019 serves as a prerequisite for conducting a cohort study. Moreover, NHANES can access the mortality data of the National Center for Health Statistics (NCHS) through an interface. Since all study participants have provided informed consent and the study has been approved by the NCHS Institutional Review Board, no additional informed consent or ethical review was necessary. All methods have been carried out in line with relevant guidelines and regulations.

**Figure 1:**
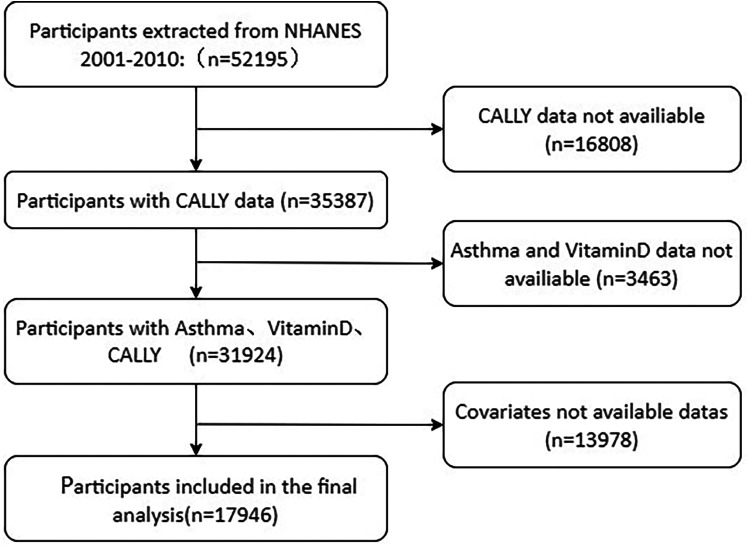
Flow diagram of selecting populations for analysis.

The study encompassed individuals aged 18 years and above who took part in the U.S. National Health Survey between 2001 and 2010. To guarantee the validity and reliability of the results, strict exclusion criteria were applied. Among the 52,195 participants in the 2001–2010 NHANES cycle, 32,874 had accessible CALLY data. Those lacking data regarding CALLY, asthma, coronary heart disease (CHD), vitamin D, and body mass index (BMI) were excluded from the study, resulting in a final study population of 17,940 participants. The study adhered to the Declaration of Helsinki, and the sample selection process is depicted in a flowchart presented in Figure 1.

### Definition of asthma

Referring to existing asthma research (9), individuals who answered “yes” to the question, “Has a doctor or other health professional told you that you have asthma?” were considered to have asthma. While this self-reported physician diagnosis has limitations, in future research, we plan to validate it with additional NHANES criteria, such as lung function test data [e.g., forced expiratory volume in 1 second (FEV₁)/forced vital capacity (FVC) ratio] and use of asthma medication. Lung function tests are important objective indicators for diagnosing asthma and can directly reflect the degree of airway obstruction, while use of asthma medication can assist in judging the diagnosis and control of asthma. By integrating these indicators, we can more accurately define asthma, reduce bias caused by self-reporting, and improve the reliability of research results (10, 11).

### Definition of CALLY

Complete blood counts were performed using a Coulter® DxH 800 analyzer under the supervision of trained medical staff. The CALLY index was calculated as albumin (g/L) × lymphocytes (109/L) × CRP (mg/L) × 10.

### Definition of serum vitamin D concentration

Serum samples from NHANES 2001–2010 were evaluated using liquid chromatography-tandem mass spectrometry and serum total vitamin D levels (nmol/L) were calculated by summing 25-hydroxyVitamin D_2_ and 25-hydroxyVitamin D_3_. Participants with missing data on serum vitamin D concentrations were excluded from the study (12).

### Ascertainment of covariates

The present study integrated multiple potential covariates. These included demographic details, living conditions, disease states, complete blood count parameters, and biochemical markers. Demographic information covered variables such as sex, age, race, marital standing, and educational attainment. Participant lifestyle aspects were determined by asking about smoking habits, alcohol intake, and calculating BMI. Disease status focused on the presence of hypertension and diabetes, as reported in the study questionnaires. Regarding the complete blood count, measurements involved leukocytes, monocytes, neutrophils, platelets, and lymphocytes. Biochemical indices under consideration were total cholesterol (TC) and aminotransferase (AST). The study investigated how these factors might potentially influence the relationship between the CALLY index and asthma. All procedures were executed following a standardized protocol.

### Statistical analyses

Statistical analyses were performed using SPSS software version 27, with a significance level set at *p* < 0.05. The statistical results were presented alongside reported odds ratios (OR) and 95% confidence intervals (CI). The CALLY dataset was divided into four equal parts. The first quartile (Q1) was assigned as the reference group. Continuous variables were presented in terms of means and standard deviations, while categorical variables were shown as frequencies or percentages. To assess differences among the CALLY quartiles, either the chi-square test or the Kruskal–Wallis *H*-test was employed. Four logistic regression models were developed to explore the association between the CALLY index and asthma. The initial model was left unadjusted. The second model underwent adjustment for variables such as gender, age, marital status, educational attainment, and presence of marriage. Building upon model 2, model 3 incorporated additional variables related to lifestyle, namely alcohol consumption and smoking habits. Subsequently, model 4 was further extended to include medical conditions like hypertension, diabetes mellitus, and total cholesterol levels. Subgroup analyses were conducted to explore potential disparities in the relationship between CALLY and asthma within specific populations, stratified by age, gender, alcohol consumption status, smoking behavior, presence of hypertension, diabetes, and other relevant factors. To ensure a more precise evaluation of the association between CALLY and asthma, correlation analyses were carried out. The results of these analyses indicated a linear negative relationship, and for the sake of brevity, were not included in the manuscript text.

Finally, through mediation analyses, we were able to determine that vitamin D exerted a mediating effect. This approach not only offers statistical evidence for mechanistic investigations but is also well-suited for uncovering underlying pathways. The direct effect demonstrated a relationship between the CALLY index and asthma. The indirect effect indicated that the association between CALLY and asthma was mediated by vitamin D levels. Moreover, the mediator ratio presented the percentage proportion of the mediating effect. By employing these statistical methods, we were able to conduct a more comprehensive exploration of the potential associations between the CALLY index and the risk of asthma. Despite the modest mediation effect of vitamin D (3.36%), it is noteworthy from a clinical perspective as it signifies the involvement of vitamin D in the association between the CALLY index and asthma. In particular, for high-risk groups with vitamin D deficiency and an abnormal CALLY index, the supplementation of vitamin D may offer a means to mitigate the risk of developing asthma (13). Concomitantly, this finding suggests the presence of unmeasured confounding factors, which may have an impact on the mediation effect size of vitamin D. Further research is necessary to explore these factors and enhance our comprehension of the etiology of asthma (14).

In addition, although lymphocytes play a pivotal role in the immune response, the lymphocyte count in this study did not demonstrate a significant association with asthma risk. This may be attributable to the multifaceted functions of lymphocyte subsets. Lymphocytes comprise a variety of subsets. The roles of these subsets in asthma are intricate and may counteract each other. For instance, Th1 and Th2 cells exert opposite effects in the asthmatic inflammatory response. Th2 cells can promote inflammation, while Th1 cells may inhibit it, and this complex interaction may lead to an unclear relationship between the overall lymphocyte count and asthma risk (15). The limitations of this study include the potential impact of sample characteristics, research design, and confounding factors on the detection of the association between lymphocyte count and asthma risk. Future research can further subdivide lymphocyte subsets to explore the relationship between each subset and asthma risk more comprehensively (16).

## Results

After applying appropriate exclusion criteria, a total of 17,946 patients were enrolled, including 9,455 men and 8,491 women. Figure 1 illustrates the process of screening participants and provides detailed information.

### Asthma-based baseline characteristics of the population

Table 1 provides a summary of the baseline characteristics of the study population, categorized according to the presence or absence of asthma. The prevalence of asthma in the overall population was found to be 12.91%, with the condition being detected in 2,317 out of a total of 17,946 participants. The mean age of participants in this study was 49 years. With respect to gender distribution, 52.69% of the participants were male and 47.31% were female. Women, individuals aged under 60 years, non-Hispanic whites, and non-drinkers exhibited a higher risk of asthma compared to other patients (all *P* < 0.05). The subsequent table (Table 2) divides participants into quartiles based on their CALLY values. Q1 corresponded to values below 1.75, Q2 corresponded to values between 1.75 and 4.20, Q3 corresponded to values between 4.20 and 10.50, and Q4 corresponded to values above 10.50. The prevalence of asthma increased with increasing CALLY values of the participants in the following order (Q1: 15.32%, Q2: 13.14%, Q3: 11.42%, Q4: 11.75%; *P* < 0.001).

**Table 1:** Baseline characteristics of the asthma group versus the non-asthma group.

Variables	Total (*n* = 17,946)	0 (*n* = 15,629)	1 (*n* = 2,317)	*P*
Age (years)	49.04 ± 18.00	49.45 ± 18.01	46.28 ± 17.65	**<0**.**001**
BMI (kg/m^2^)	28.69 ± 6.48	28.50 ± 6.29	29.94 ± 7.51	**<0**.**001**
Vitamin D (nmol/L)	63.01 ± 23.45	63.17 ± 23.37	61.89 ± 23.93	**0**.**014**
ALB (g/dL)	4.22 ± 0.36	4.22 ± 0.35	4.18 ± 0.37	**<0**.**001**
AST (U/L)	25.82 ± 19.19	25.86 ± 19.91	25.54 ± 13.34	0.454
TC (mg/dL)	199.65 ± 42.09	200.07 ± 42.00	196.81 ± 42.59	**<0**.**001**
WBC (1,000 cells/µL)	7.31 ± 2.64	7.29 ± 2.68	7.50 ± 2.33	**<0**.**001**
Lymphocytenumber (1,000 cells/µL)	2.15 ± 1.42	2.15 ± 1.49	2.17 ± 0.80	0.527
Monocytenumber (1,000 cells/µL)	0.56 ± 0.20	0.56 ± 0.20	0.55 ± 0.19	0.348
Neutrophilsnumber (1,000 cells/µL)	4.36 ± 1.88	4.34 ± 1.89	4.49 ± 1.86	**<0**.**001**
Plateletcount (1,000 cells/µL)	261.34 ± 69.55	260.46 ± 69.54	267.29 ± 69.34	**<0**.**001**
CRP (mg/dL)	0.44 ± 0.86	0.43 ± 0.87	0.50 ± 0.77	**<0**.**001**
CALLY	10.03 ± 22.80	10.19 ± 23.66	8.96 ± 15.81	**0**.**016**
Gender, *n* (%)				**<0**.**001**
Male	9,455 (52.69)	8,411 (53.82)	1,044 (45.06)	
Female	8,491 (47.31)	7,218 (46.18)	1,273 (54.94)	
Race, *n* (%)				**<0**.**001**
Mexican American	3,385 (18.86)	3,148 (20.14)	237 (10.23)	
Others	1,731 (9.65)	1,468 (9.39)	263 (11.35)	
Non-Hispanic White	9,603 (53.51)	8,274 (52.94)	1,329 (57.36)	
Non-Hispanic Black	3,227 (17.98)	2,739 (17.53)	488 (21.06)	
Education, *n* (%)				**<0**.**001**
High school and below	9,078 (50.59)	7,995 (51.15)	1,083 (46.74)	
Some college or above	8,868 (49.41)	7,634 (48.85)	1,234 (53.26)	
Marital, *n* (%)				**<0**.**001**
Partner	11,342 (63.20)	10,021 (64.12)	1,321 (57.01)	
No partner	6,604 (36.80)	5,608 (35.88)	996 (42.99)	
Drinking, *n* (%)				**0**.**003**
Yes	2,995 (16.69)	2,559 (16.37)	436 (18.82)	
No	14,951 (83.31)	13,070 (83.63)	1,881 (81.18)	
Smoking, *n* (%)				**<0**.**001**
Yes	9,581 (53.39)	8,261 (52.86)	1,320 (56.97)	
No	8,365 (46.61)	7,368 (47.14)	997 (43.03)	
Hypertension, *n* (%)				**<0**.**001**
Yes	5,808 (32.36)	4,936 (31.58)	872 (37.63)	
No	12,138 (67.64)	10,693 (68.42)	1,445 (62.37)	
Diabetes, *n* (%)				**<0**.**001**
Yes	1,851 (10.31)	1,562 (9.99)	289 (12.47)	
No	16,095 (89.69)	14,067 (90.01)	2,028 (87.53)	

Continuous and categorical variables are displayed individually as mean ± SD or proportions. Bold values indicate statistical significance (*P* < 0.05).

HR, hazard ratio; CI, confidence interval; BMI, body mass index; TC, total cholesterol; CALLY, C-reactive protein-albumin-lymphocyte.

**Table 2:** Baseline characteristics of the study population according to the CALLY index in NHANES 2001–2010.

Variables	Total (*n* = 17,946)	1 (*n* = 4,479)	2 (*n* = 4,535)	3 (*n* = 4,438)	4 (*n* = 4,494)	*P*
Age (years)	49.04 ± 18.00	51.20 ± 18.22	51.54 ± 17.81	49.57 ± 17.64	43.82 ± 17.23	**<0**.**001**
BMI (kg/m^2^)	28.69 ± 6.48	31.99 ± 7.91	29.65 ± 5.97	27.81 ± 5.14	25.31 ± 4.38	**<0**.**001**
Vitamin D (nmol/L)	63.01 ± 23.45	60.58 ± 24.26	62.12 ± 23.02	64.26 ± 23.30	65.09 ± 22.94	**<0**.**001**
ALB (g/dL)	4.22 ± 0.36	3.97 ± 0.38	4.19 ± 0.30	4.30 ± 0.30	4.41 ± 0.29	**<0**.**001**
AST (U/L)	25.82 ± 19.19	25.72 ± 30.01	25.94 ± 16.34	26.15 ± 12.57	25.46 ± 12.14	0.374
TC (mg/dL)	199.65 ± 42.09	201.14 ± 43.79	203.24 ± 43.26	201.19 ± 41.22	193.02 ± 39.19	**<0**.**001**
WBC (1,000 cells/µL)	7.31 ± 2.64	7.79 ± 2.78	7.31 ± 2.36	7.13 ± 2.03	7.02 ± 3.16	**<0**.**001**
Lymphocytenumber (1,000 cells/µL)	2.15 ± 1.42	1.96 ± 0.68	2.11 ± 0.88	2.18 ± 0.80	2.35 ± 2.46	**<0**.**001**
Monocytenumber (1,000 cells/µL)	0.56 ± 0.20	0.58 ± 0.21	0.56 ± 0.19	0.55 ± 0.19	0.54 ± 0.21	**<0**.**001**
Neutrophilsnumber (1,000 cells/µL)	4.36 ± 1.88	5.00 ± 2.36	4.39 ± 1.87	4.15 ± 1.54	3.89 ± 1.44	**<0**.**001**
Plateletcount (1,000 cells/µL)	261.34 ± 69.55	275.08 ± 78.60	261.76 ± 67.39	256.63 ± 66.07	251.87 ± 63.00	**<0**.**001**
CRP (mg/dL)	0.44 ± 0.86	1.24 ± 1.43	0.33 ± 0.15	0.15 ± 0.07	0.05 ± 0.04	**<0**.**001**
Gender, *n* (%)						**<0**.**001**
Male	9,455 (52.69)	1,862 (41.57)	2,289 (50.47)	2,602 (58.63)	2,702 (60.12)	
Female	8,491 (47.31)	2,617 (58.43)	2,246 (49.53)	1,836 (41.37)	1,792 (39.88)	
Race, *n* (%)						**<0**.**001**
Mexican American	3,385 (18.86)	815 (18.20)	866 (19.10)	891 (20.08)	813 (18.09)	
Others	1,731 (9.65)	362 (8.08)	413 (9.11)	441 (9.94)	515 (11.46)	
Non-Hispanic White	9,603 (53.51)	2,327 (51.95)	2,436 (53.72)	2,393 (53.92)	2,447 (54.45)	
Non-Hispanic Black	3,227 (17.98)	975 (21.77)	820 (18.08)	713 (16.07)	719 (16.00)	
Education, *n* (%)						**<0**.**001**
High school and below	9,078 (50.59)	2,413 (53.87)	2,333 (51.44)	2,264 (51.01)	2,068 (46.02)	
Some college or above	8,868 (49.41)	2,066 (46.13)	2,202 (48.56)	2,174 (48.99)	2,426 (53.98)	
Marital, *n* (%)						**<0**.**001**
Partner	11,342 (63.20)	2,722 (60.77)	2,893 (63.79)	2,960 (66.70)	2,767 (61.57)	
No partner	6,604 (36.80)	1,757 (39.23)	1,642 (36.21)	1,478 (33.30)	1,727 (38.43)	
Drinking, *n* (%)						0.082
Yes	2,995 (16.69)	730 (16.30)	813 (17.93)	722 (16.27)	730 (16.24)	
No	14,951 (83.31)	3,749 (83.70)	3,722 (82.07)	3,716 (83.73)	3,764 (83.76)	
Smoking, *n* (%)						**<0**.**001**
Yes	9,581 (53.39)	2,510 (56.04)	2,419 (53.34)	2,387 (53.79)	2,265 (50.40)	
No	8,365 (46.61)	1,969 (43.96)	2,116 (46.66)	2,051 (46.21)	2,229 (49.60)	
Hypertension, *n* (%)						**<0**.**001**
Yes	5,808 (32.36)	1,882 (42.02)	1,605 (35.39)	1,359 (30.62)	962 (21.41)	
No	12,138 (67.64)	2,597 (57.98)	2,930 (64.61)	3,079 (69.38)	3,532 (78.59)	
Diabetes, *n* (%)						**<0**.**001**
Yes	1,851 (10.31)	631 (14.09)	510 (11.25)	406 (9.15)	304 (6.76)	
No	16,095 (89.69)	3,848 (85.91)	4,025 (88.75)	4,032 (90.85)	4,190 (93.24)	
Asthma, *n* (%)						**<0**.**001**
No	15,629 (87.09)	3,793 (84.68)	3,939 (86.86)	3,931 (88.58)	3,966 (88.25)	
Yes	2,317 (12.91)	686 (15.32)	596 (13.14)	507 (11.42)	528 (11.75)	

Continuous and categorical variables are displayed individually as mean ± SD or proportions. Bold values indicate statistical significance (*P* < 0.05).

HR, hazard ratio; CI, confidence interval; BMI, body mass index; TC, total cholesterol; CALLY, C-reactive protein-albumin-lymphocyte.

### Logistic regression analyses

The associations between CALLY and asthma, vitamin D, and asthma are shown in Table 3, which was obtained from multivariate logistic regression analyses. In the original model, CALLY was significantly negatively associated with asthma, with a contrast ratio of 0.996 (95% CI: 0.993–0.999; *P* = 0.006). The robust and favorable association in model 2 was still highly significant (OR = 0.994, 95% CI: 0.991–0.997; *P* < 0.001) even after taking into account factors such as gender, age, marriage, race, and education. The association between CALLY and asthma remained statistically significant (OR = 0.994, 95% CI: 0.991–0.997; *P* < 0.001) in model 3, which was developed on the basis of model 2. Table 3 shows the contrast ratio and 95% CIs according to CALLY and asthma, even after considering variables such as smoking habits and alcohol consumption. The aforementioned significant correlation remained statistically significant (OR = 0.995, 95% CI: 0.992–0.998; *P* < 0.001) even when model 4 was expanded to include hypertension, diabetes, and total cholesterol. This indicates that a 1-unit increase in CALLY is associated with a 0.995-fold decrease in asthma. After the division of CALLY into quartiles, the correlation between CALLY and asthma remained, and the test for trend was statistically significant (*P* < 0.05). In the fourth model, the highest quartile of CALLY was compared with the lowest quartile, and a 1-unit increase in CALLY was associated with a 0.75-fold decrease in asthma. Multimodel regression analyses of the three components of CALLY were also performed, and it was found that CPR had a significant positive association with the development of asthma, albumin had a significant negative association with the development of asthma, and lymphocytes did not show significance and did not have an effect on asthma. In model 4, controlling for confounders, a 1-unit increase in CPR was associated with a 1.05-fold increase in asthma, and a 1-unit increase in albumin (g/dL) was associated with a 0.8-fold decrease in asthma. In addition, we found a negative but not significant association between vitamin D and asthma. Therefore, we performed quartiles of vitamin D, comparing the highest quartile of vitamin D with the lowest quartile, and found that asthma decreased 1.05-fold when vitamin D was increased by 1 unit.

**Table 3:** Odds ratios and 95% confidence intervals for asthma according to the CALLY index and vitamin D.

Exposure	OR (95% CI), *P*-value			
	Model 1	Model 2	Model 3	Model 4
CALLY (continuous)	0.996 (0.993–0.999)0.006	0.994 (0.991–0.997) < .001	0.994 (0.991–0.997) < .001	0.995 (0.992–0.998) < .001
CALLY (quartile)				
Quartile 1	Reference	Reference	Reference	Reference
Quartile 2	0.84 (0.74–0.94) 0.003	0.87 (0.77–0.98) 0.018	0.87 (0.77–0.98) 0.021	0.90 (0.80–1.02) 0.097
Quartile 3	0.71 (0.63–0.81) < .001	0.74 (0.66–0.84) < .001	0.75 (0.66–0.85) < .001	0.79 (0.70–0.90) < .001
Quartile 4	0.74 (0.65–0.83) < .001	0.70 (0.62–0.80) < .001	0.71 (0.63–0.81) < .001	0.75 (0.66–0.86) < .001
Vitamin D (continuous)	0.998 (0.996–1.000) 0.014	0.999 (0.997–1.000) < .001	0.998 (0.997–1.000) < .001	0.999 (0.997–1.001) 0.004
Vitamin D (quartile)				
Quartile 1	Reference	Reference	Reference	Reference
Quartile 2	0.88 (0.78––0.99) 0.041	0.93 (0.82–1.06) 0.26	0.94 (0.82–1.06) 0.308	0.94 (0.83–1.07) 0.383
Quartile 3	0.79 (0.70–0.90) < .001	0.81 (0.71–0.92) 0.002	0.81 (0.71–0.93) 0.003	0.83 (0.73–0.95) 0.008
Quartile 4	0.86 (0.76–0.97) 0.016	0.79 (0.69–0.91) < .001	0.79 (0.69–0.91) 0.001	0.82 (0.71–0.94) 0.005
CRP	1.07 (1.03–1.12) < .001	1.07 (1.03–1.12) 0.001	1.07 (1.02–1.12) 0.003	1.05 (1.01–1.10) 0.034
ALB	0.71 (0.63–0.80) < .001	0.75 (0.66–0.85) < .001	0.76 (0.67–0.86) < .001	0.80 (0.70–0.91) < .001
Lymphocyte number	1.01 (0.98–1.04) 0.529	1.00 (0.97–1.04) 0.797	1.00 (0.96–1.03) 0.901	0.99 (0.96–1.03) 0.761

Continuous and categorical variables are displayed individually as mean ± SD or proportions. Model 1: no adjustment for covariates. Model 2: adjusted for sex, age, race, education level, marital status. Model 3: adjusted for sex, age, race, education level, marital status, and smoking status, drinking. Model 4: adjusted for sex, age, race, education level, marital status, and smoking status, drinking, hypertension, diabetes, and TC.

HR, hazard ratio; CI, confidence interval; BMI, body mass index; TC, total cholesterol; CALLY, C-reactive protein-albumin-lymphocyte.

The results of the linear logistic regression analyses examining the relationship between CALLY and vitamin D are shown in Table 4. In the unadjusted model, CALLY demonstrated a significant and positive correlation with vitamin D (OR = 0.035, 95% CI: 0.020–0.050; *P* < 0.001). This positive association remained significant even after controlling for sex, age, marital status, race, and other relevant variables in model 2 (OR = 0.044, 95% CI: 0.029–0.059; *P* < 0.001). Similar to model 2, model 3 adjusted for smoking habits and alcohol consumption based on model 2, and the correlation between CALLY and vitamin D remained significant (OR = 0.045, 95% CI: 0.03–0.06; *P* < 0.001). These consistent results across different models suggest that the relationship between CALLY and vitamin D is robust and not easily confounded by various factors. Moreover, the previously mentioned positive association remained statistically significant even after controlling for variables in model 4 (OR = 0.043, 95% CI: 0.028–0.058; *P* < 0.001). The division of CALLY into quartiles resulted in a statistically significant positive effect relationship on vitamin D, and the test for trend was statistically significant (*P* < 0.010). In addition, elevated levels of CALLY were observed to exert a significant positive influence on vitamin D levels in model 4, particularly when the highest quartile of CALLY was juxtaposed with the lowest quartile. Furthermore, multimodel regression analyses of the three components of CALLY revealed a significant negative correlation between CPR and lymphocytes with vitamin D, and a significant positive correlation between albumin and vitamin D. Consequently, vitamin D levels exhibited a significant positive correlation with the CALLY index, indicating that elevated vitamin D levels are associated with higher CALLY index values.

**Table 4:** Multiple logistic regression of the association between the CALLY index and asthma mellitus.

Exposure	OR (95% CI), *P*-value			
	Model 1	Model 2	Model 3	Model 4
CALLY (continuous)	0.035 (0.020–0.050) < .001	0.044 (0.029–0.059) < .001	0.045 (0.03–0.06) < .001	0.043 (0.028–0.058) < .001
CALLY (quartile)				
Quartile 1	Reference	Reference	Reference	Reference
Quartile 2	1.535 (0.569–2.5) *P* < 0.002	1.432 (0.476–2.387) *P* < 0.003	1.486 (0.531–2.442) *P* < 0.002	1.142 (0.189–2.095) *P* < 0.019
Quartile 3	3.676 (2.705–4.647) *P* < 0.000	3.748 (2.78–4.716) *P* < 0.000	3.811 (2.844–4.779) *P* < 0.000	3.377 (2.41–4.343) *P* < 0.000
Quartile 4	4.504 (3.536–5.472) *P* < 0.000	5.162 (4.185–6.139) *P* < 0.000	5.254 (4.276–6.232) *P* < 0.000	4.905 (3.924–5.885) *P* < 0.000
CRP	−1.055 (−1.47–0.64) *P* < 0.000	−0.916 (−1.326–0.506) *P* < 0.000	−0.944 (−1.354–0.535) *P* < 0.000	−0.846 (−1.254–0.437) *P* < 0.000
ALB	4.921 (3.918–5.924) *P* < 0.000	6.408 (5.362–7.453) *P* < 0.000	6.457 (5.412–7.502) *P* < 0.000	5.95 (4.906–6.993) *P* < 0.000
Lymphocyte number	−0.553 (−0.794–0.312) *P* < 0.000	−0.478 (−0.716–0.239) *P* < 0.000	−0.507 (−0.746–0.269) *P* < 0.000	−0.493 (−0.731–0.255) *P* < 0.000

Continuous and categorical variables were displayed individually as mean ± SD or proportions. Model 1: no adjustment for covariates. Model 2: adjusted for sex, age, race, education level, marital status. Model 3: adjusted for sex, age, race, education level, marital status, and smoking status, drinking. Model 4: adjusted for sex, age, race, education level, marital status, and smoking status, drinking, hypertension, diabetes, and TC.

HR, hazard ratio; CI, confidence interval; BMI, body mass index; TC, total cholesterol; CALLY, C-reactive protein-albumin-lymphocyte.

### Subgroup analyses and interaction tests

To assess the stability of the association between CALLY and asthma, and to identify potential differences between different populations, a series of subgroup analyses were performed, the results of which are shown in Table 5. It is notable that the association between CALLY and high asthma risk was more pronounced among participants in the age ≥60 years, female, alcohol, smoking, hypertension, and diabetes subgroups (all *P*-values < 0.05). Conversely, the “*P* for interaction” was greater than 0.05 in the reciprocal test, suggesting that the negative association between CALLY and asthma is generally stable and consistent in the general population.

**Table 5:** Subgroups analysis for the associations between the CALLY index and asthma.

Variables	*n* (%)	OR (95% CI)	*P*	*P* for interaction
All patients	17,946 (100.00)	0.90 (0.86–0.93)	**<0**.**001**	
Gender				0.576
Male	9,455 (52.69)	0.93 (0.87–0.98)	**0**.**009**	
Female	8,491 (47.31)	0.90 (0.86–0.95)	**<0**.**001**	
Drinking				0.669
Yes	2,995 (16.69)	0.88 (0.80–0.96)	**0**.**006**	
No	14,951 (83.31)	0.90 (0.86–0.94)	**<0**.**001**	
Smoking				0.528
Yes	9,581 (53.39)	0.89 (0.84–0.94)	**<0**.**001**	
No	8,365 (46.61)	0.91 (0.86–0.97)	**0**.**002**	
Hypertension				0.090
Yes	5,808 (32.36)	0.87 (0.81–0.93)	**<0**.**001**	
No	12,138 (67.64)	0.93 (0.89–0.98)	**0**.**005**	
Diabetes				<0.001
Yes	1,851 (10.31)	0.72 (0.64–0.82)	**<0**.**001**	
No	16,095 (89.69)	0.92 (0.89–0.96)	**<0**.**001**	
Age				0.329
Below 60	12,186 (67.90)	0.89 (0.85–0.93)	**<0**.**001**	
Over 60	5,760 (32.10)	0.85 (0.78–0.92)	**<0**.**001**	

Bold values indicate statistical significance (*P* < 0.05).

### Mediation of vitamin D

Mediation analyses were conducted by creating three pathways (a, b, and c). For this purpose, we set up three pathways: (1) exposure to mediation; (2) mediation to outcome (direct effect); and (3) exposure to outcome (total effect). The total effect reflects the sum of the direct and mediated (indirect) effects. The percentage of mediated effects was calculated using the following formula: (mediated effect/total effect) × 100 (17).

**Figure 2:**
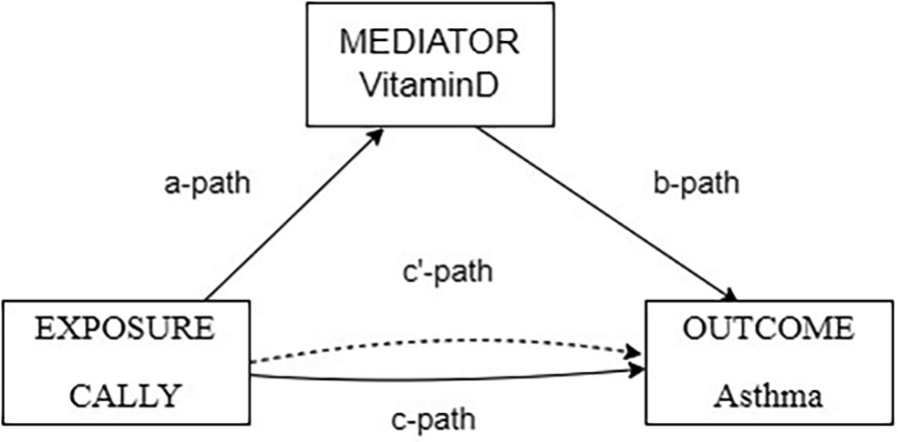
Path diagram of the mediation analysis models.

Mediation analyses were performed to assess whether vitamin D mediated the association between CALLY and asthma. The model and pathway for the intermediary analysis are shown in Figure 2. The results of the intermediary analysis showed that CALLY had an indirect effect on asthma development in patients with vitamin D (B: 0.000, 95% CI: 0.000–0.000; *P* = 0.976), suggesting that vitamin D partially mediated the effect. The effect of CALLY on asthma was still statistically significant when controlling for vitamin D (B: 0.000, 95% CI: 0.000–0.000; *P* = 0.020), suggesting both direct and indirect effects. Approximately 3.36% of the effects of CALLY on asthma were mediated by vitamin D. The results of mediation analyses are shown in Table 6.

**Table 6:** Mediation analysis for the associations between the CALLY index and asthma.

Independent variable mediator	Mediator	Total effect		Indirect effect		Direct effect		Proportion mediated, %(95% CI)
		Coefficient (95% CI)	*P*-value	Coefficient (95% CI)	*P*-value	Coefficient (95% CI)	*P*-value	
CALLY	VitaminD	−0.000 (−0.000–−0.000)	0.016	−0.000 (−0.000–−0.000)	0.976	−0.000 (−0.000–−0.000)	0.02	3.36

In mediation analyses, adjustments were made for sex, age, race, education level, marital status, BMI, smoking status, drinking, hypertension, diabetes, and TC.

BMI, body mass index; TC, total cholesterol; CALLY, C-reactive protein-albumin-lymphocyte.

### Correlation between CALLY values and asthma and vitamin D-related measurements

**Figure 3:**
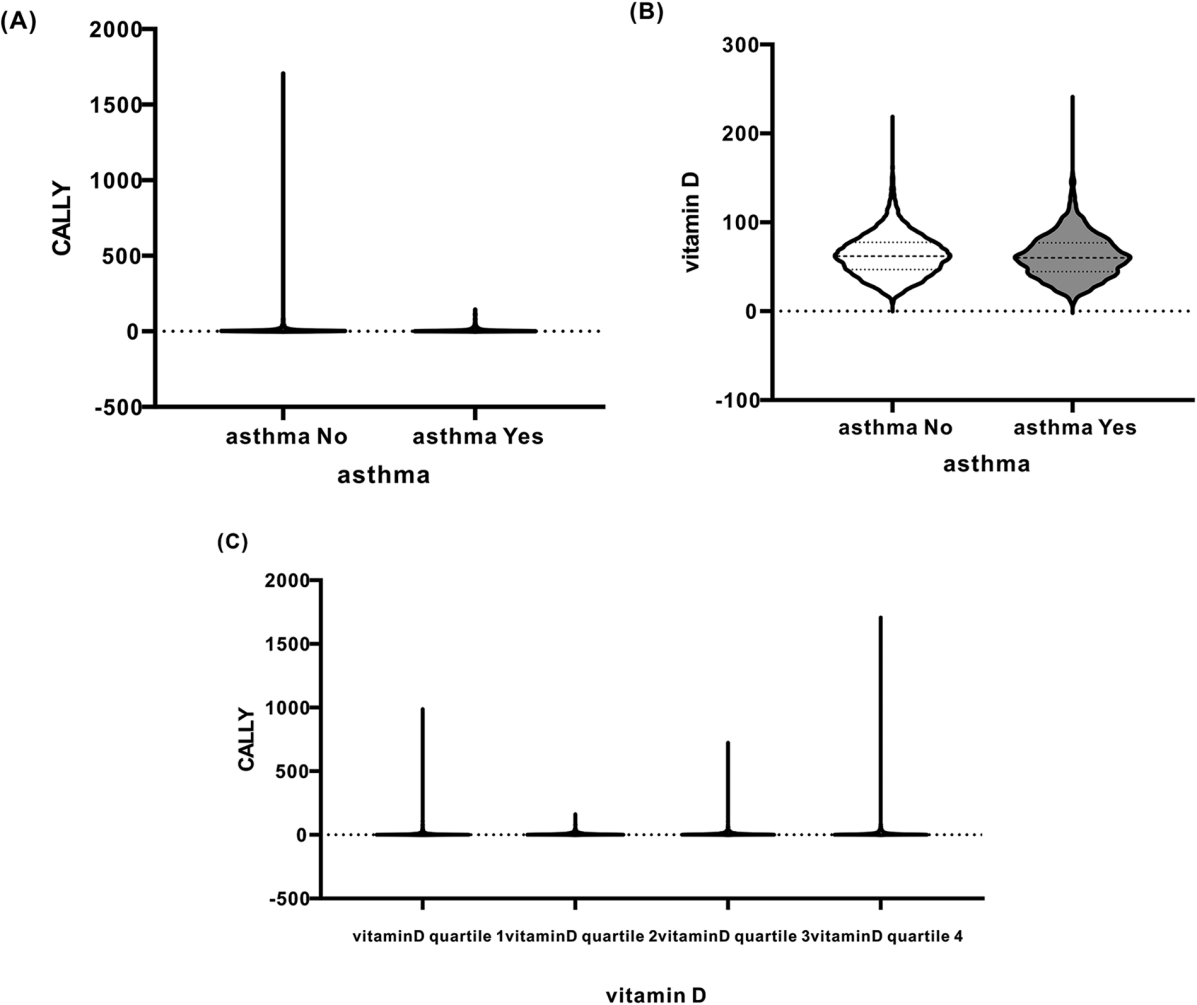
**(A)** Relationship between CALLY (*Y*-axis) and asthma (asthma, *X*-axis). **(B)** Relationship between vitamin D (*Y*-axis) and asthma (asthma, *X*-axis). **(C)** Relationship between CALLY (*Y*-axis) and vitamin D quartile (*X*-axis).

Spearman rank correlation analyses were used to examine the relationship between the CALLY index distribution and asthma measures, while violin plots visualized the distribution of vitamin D levels. There was a significant negative correlation between CALLY values and asthma, and a significant positive correlation between CALLY values and vitamin D. The correlation between CALLY values and vitamin D was also significant. Similarly, the violin plot demonstrated the correlation between CALLY values and asthma measures and vitamin D in Figures 3A–C.

## Discussion

This research represents the initial report on the mediating function of vitamin D within the relationship between the CALLY index and asthma. Data from 17,946 adults were analyzed. A significant correlation was detected between the CALLY index and asthma, indicating that those with an elevated CALLY index are less prone to developing asthma. Even after adjusting for multiple covariates in the fully adjusted model 4, this correlation persisted robustly. Subgroup analyses and interaction tests demonstrated that the inverse association between the CALLY index and asthma was more prominent among participants who were aged 60 years old or older, female, consumed alcohol, smoked cigarettes, had hypertension, or had diabetes mellitus.

The interactions among these subgroups were determined to be non-significant and relatively stable. Overall, the findings of this study demonstrate a negative correlation between the CALLY index and asthma. Simultaneously, the results indicate that vitamin D exerts a mediating effect on the association between the CALLY index and asthma. This highlights the importance of taking into account both inflammatory markers, such as the CALLY index and vitamin D levels. Consequently, it underscores the need for improved management of patients with low vitamin D levels as a preventive measure against asthma. By doing so, healthcare providers can potentially mitigate the risk of asthma development, especially in individuals with suboptimal vitamin D status, and better understand the complex interplay between inflammation and vitamin D in the context of asthma pathogenesis.

Asthma ranks as the second most prevalent non-communicable chronic respiratory disorder globally. It is defined by airflow obstruction, which results in breathlessness and wheezing (18). Alarmingly, the under-diagnosis and under-treatment of asthma among both children and adults pose substantial public health challenges (19, 20). Inflammation is recognized as a pivotal factor in the pathogenesis and progression of asthma (21). Ample evidence (22–24) indicates that airway inflammation is a fundamental aspect of asthma. It is linked to bronchial hyperresponsiveness and impacts disease severity. The inflammatory process in asthma is intrinsically multicellular, involving mast cells, neutrophils, eosinophils, T-lymphocytes, and epithelial cells in the immune response.

To this day, no research has specifically investigated the prevalence of the CALLY index in relation to asthma, nor has any study explored whether serum vitamin D levels play a mediating role in this relationship.

The CALLY index, calculated from the combined assessment of lymphocyte count, serum albumin, and CRP levels, represents a novel and comprehensive metric for evaluating nutritional and inflammatory states. Numerous clinical investigations have identified an inverse correlation between CRP and lung function parameters, such as the forced expiratory volume in 1 s to forced vital capacity ratio (FEV₁/FVC) (25, 26). In addition, elevated CRP levels are associated with reduced responsiveness to treatment and more significant declines in lung function among asthma patients. As a result, CRP has emerged as one of the most crucial indices for assessing the condition and prognosis of asthma. Typically used as a static measurement, CRP is correlated with disease severity and prognosis (27, 28). Jun You (29) identified the potential of the CALLY index as a valuable biomarker for predicting asthma prevalence and mortality, highlighting its significance in asthma management and prognosis assessment. Hypoalbuminemia has been shown to affect the body's immune system. It leads to suppression of immune cells and a decrease in the synthesis of immune factors. Consequently, this weakens the body's resistance against asthma-related pathogens and promotes the development of asthma. In addition, hypoalbuminemia might be associated with airway repair and remodeling processes in asthma. Albumin serves as a crucial indicator for evaluating nutritional status. Studies have demonstrated that compared to the non-asthmatic population, individuals with asthma have lower serum albumin levels (30). This difference is also evident in asthmatic children (31).

A study observed an inverted U-shaped relationship between serum albumin and bronchial eosinophilic inflammation in asthma (BEOC), indicating a potential connection between the overall nutritional state and immune system alterations in asthma patients (32). In another investigation, Rongjuan Zhuang discovered a negative linear correlation between serum albumin levels and all-cause mortality among asthmatic individuals (33). It has been shown that declining renal function can decrease serum albumin levels and its ability to bind uremic toxins (34). Furthermore, immune cells have been proven to be crucial in both cardiac injury processes and subsequent repair mechanisms (35, 36).

Activated lymphocytes are a key element in the airway inflammation characteristic of asthma. These lymphocytes can interact with other immune cells, giving rise to a complex immunoregulatory network. In the context of asthma, when this immune network is disrupted, it not only impacts the initiation and sustenance of the inflammatory response. It also engages with airway structural cells and contributes to the airway remodeling process, thereby influencing the long-term prognosis of asthma. Therefore, the CALLY index can be considered a more responsive metric that reflects the systemic immune and inflammatory reactions.

Zhehao Xu's research findings suggest that the CALLY index can function as an independent predictor of the risk of cardiorenal syndrome (CRS). It demonstrates greater predictive efficiency compared to other inflammation-based composite indices, such as the Systemic Immune-Inflammation Index (SII), Neutrophil-to-High-density lipoprotein cholesterol Ratio (NHR), Lymphocyte-to-High-density lipoprotein cholesterol Ratio (LHR), Monocyte-to-High-density lipoprotein cholesterol Ratio (MHR), and Platelet-to-High-density lipoprotein cholesterol Ratio (PHR). The CALLY index reflects the inflammatory, immune, and nutritional status of patients. Currently, it is primarily applied in the diagnosis of oncology patients. Moreover, it has emerged as a novel and promising prognostic biomarker for breast, oral, colorectal, and gastric cancers (37–40). Studies have uncovered a notable negative correlation between the CALLY index and both all-cause mortality and cardiovascular mortality among the elderly (41). Moreover, a retrospective, single-center observational study has determined that the CALLY index serves as an independent risk predictor for acute kidney injury (AKI) episodes (42). These research findings offer indirect support for our results. They imply that the CALLY index, being a composite marker, can offer comprehensive prognostic details. This indicates its potential value in assessing various health outcomes, similar to how it may play a role in the relationship being studied, such as its connection to asthma as in our current research.

The relationship between vitamin D and asthma has been extensively studied. Many studies suggest that low vitamin D levels are linked to a higher risk of developing asthma and more severe symptoms in asthmatic individuals.

Vitamin D is involved in multiple physiological processes, especially immune regulation. It binds to the vitamin D receptor on immune cells, regulating their differentiation, proliferation, and function. Specifically, vitamin D can reduce airway inflammation in asthma. It affects macrophages and dendritic cells, decreasing their antigen-presenting ability and the secretion of inflammatory factors, thus preventing immune over-reaction in asthma. This leads to inhibited secretion of pro-inflammatory factors and reduced recruitment and activation of inflammatory cells in the airways, alleviating airway inflammation. Meanwhile, it promotes the production of anti-inflammatory factors, enhancing the body's anti-inflammatory capacity and maintaining the stability of the airway immune microenvironment. Since vitamin D can influence inflammatory and immune indicators related to the CALLY index, it likely plays a mediating role in the relationship between the CALLY index and asthma.

In our research, we revealed the correlation between the CALLY index and asthma. This discovery was validated by four integrated regression models. Significantly, in model 4, after accounting for confounding factors, the positive correlation between the CALLY index and vitamin D persisted significantly (OR = 0.043, 95% CI: 0.028–0.058; *P* < 0.001).

The study's notable strength lies in its utilization of a sample consisting of US adults, obtained through a meticulously stratified, multistage probability sampling method, thereby enhancing the precision of the findings. Furthermore, the implementation of four models to account for confounding variables has led to the attainment of more reliable results. Furthermore, subgroup analyses and mediation effect analyses were performed to gain more insight into the association between CALLY and asthma in different populations and the mediating role played by vitamin D between the two.

## Conclusion

The present study postulates the hypothesis that higher CALLY levels are linked to a reduced likelihood of asthma development, with vitamin D acting as a mediator. In asthma patients, the CALLY index is closely associated with vitamin D levels. Vitamin D deficiency might contribute to asthma development by leading to a low CALLY index. Our research shows that vitamin D has a crucial role in the pathophysiology of asthma. It modulates the components of the CALLY index via various mechanisms, thereby influencing the body's inflammatory and immune states. This discovery offers a new perspective on the pathogenesis of asthma.

Moreover, it suggests that vitamin D could be a potential target for preventing and treating asthma. Such a finding may lend more robust support to precision medicine research for asthma and its clinical applications.

## Data Availability

The raw data supporting the conclusions of this article will be made available by the authors, without undue reservation.
